# Editorial: Prospective utilization and clinical applications of artificial intelligence and data-driven automation for radiotherapy

**DOI:** 10.3389/fonc.2024.1445048

**Published:** 2024-07-18

**Authors:** Michael Roumeliotis, Xun Jia, Ellen Kim, Sarah Quirk

**Affiliations:** ^1^ The Johns Hopkins Hospital, Johns Hopkins Medicine, Baltimore, MD, United States; ^2^ Brigham and Women’s Hospital, Harvard Medical School, Boston, MA, United States

**Keywords:** artificial intelligence, prospective, implementation, automation, outcomes

The development of automation and artificial intelligence (AI) software has been progressing rapidly for many years in radiotherapy. These technologies have the capability to vastly improve efficiency, quality, and consistency across the entire radiotherapy treatment process as well as the ability to better understand patient outcomes (1–3). To fully realize the potential of AI and automation, overcoming the barrier of translating these advancements from ‘benchtop to bedside’ is essential. Integrating AI presents challenges similar to those encountered with other technologies in the broader radiotherapy community, such as stakeholder buy-in, practitioner training, and managing process change. However, AI also raises additional concerns, such as the interpretability of results and the potential to integrate bias in the models (4). An important intermediate step to increase the uptake of AI and automation-based software is demonstrating the validity in the prospective setting and ensuring the quality of the retrospective model is reproducible and interpretable for clinicians (5, 6). Implementing these tools prospectively within the multi-institutional settings is crucial to familiarizing clinical staff with their operation and demonstrating real-world application.

There have been successes in this space within radiotherapy, including treatment planning, brachytherapy, image analysis, and prospectively modeling patient outcomes (7–9). These examples are limited compared to the extensive studies that have performed retrospective model building applied to internal datasets or a hold-out set. The community is approaching a critical point in development where novelty is demonstrated by advancing beyond initial development and towards clinical integration. In this Research Topic of Frontiers in Oncology, we highlight works with a specific focus on the demonstration of the prospective utilization of automation and AI in clinical radiotherapy.

In this Research Topic, the articles are diverse in the specific clinical application of the automation or AI investigation but are unified in developing and validating tools that improve quality, consistency, and efficiency in the radiotherapy workflow. New commercial technologies are available, including adaptive planning workflows, AI-based contouring, and automated quality assurance, which require clinical validation before the impact can be fully realized. Three separate studies in this Research Topic authored by Kehayias et al., Galand et al., and Doolan et al., demonstrate to the community frameworks for validation and specific clinical implementations that can be emulated in future works. Separately, the development of tools for decision support in diagnosis, improving treatment quality, and better understanding patient outcomes are reported by Wang et al., Kowalchuk et al., Gan et al., and Schröder et al.. These studies demonstrate this principle in different applications through the entire treatment process, including pre-treatment lesion detection through to post-treatment survival prediction.

The highlights of these articles are intended to motivate the community to further investigate the translational steps of automation and AI tools that require validation to maximize their effect on clinical workflows and the outcomes of patients undergoing radiotherapy treatments. We anticipate that readers will find the articles both informative, motivating, and thought-provoking.

## Author contributions

MR: Conceptualization, Investigation, Methodology, Project administration, Visualization, Writing – original draft, Writing – review & editing. XJ: Conceptualization, Investigation, Writing – original draft, Writing – review & editing. EK: Investigation, Writing – original draft, Writing – review & editing. SQ: Conceptualization, Investigation, Writing – original draft, Writing – review & editing, Methodology, Project administration, Visualization.
